# Inferring an animal’s environment through biologging: quantifying the environmental influence on animal movement

**DOI:** 10.1186/s40462-020-00228-4

**Published:** 2020-10-19

**Authors:** J. A. J. Eikelboom, H. J. de Knegt, M. Klaver, F. van Langevelde, T. van der Wal, H. H. T. Prins

**Affiliations:** 1 Wildlife Ecology and Conservation Group, Wageningen University and Research, Droevendaalsesteeg 3a, 6708 PB Wageningen, Netherlands; 2 School of Life Sciences, Westville Campus, University of KwaZulu-Natal, Durban, 4000 South Africa; 3 Spatial Knowledge Systems, Wageningen Environmental Research, Droevendaalsesteeg 3a, 6708 PB Wageningen, Netherlands; 4 Department of Animal Sciences, Wageningen University and Research, De Elst 1, 6708 WD Wageningen, Netherlands

**Keywords:** Behaviour classification, Collective movement, Cows, Foraging, Group dynamics, Lactation, Machine learning, Random forest regression, Resource availability, Support vector machine

## Abstract

**Background:**

Animals respond to environmental variation by changing their movement in a multifaceted way. Recent advancements in biologging increasingly allow for detailed measurements of the multifaceted nature of movement, from descriptors of animal movement trajectories (e.g., using GPS) to descriptors of body part movements (e.g., using tri-axial accelerometers). Because this multivariate richness of movement data complicates inference on the environmental influence on animal movement, studies generally use simplified movement descriptors in statistical analyses. However, doing so limits the inference on the environmental influence on movement, as this requires that the multivariate richness of movement data can be fully considered in an analysis.

**Methods:**

We propose a data-driven analytic framework, based on existing methods, to quantify the environmental influence on animal movement that can accommodate the multifaceted nature of animal movement. Instead of fitting a simplified movement descriptor to a suite of environmental variables, our proposed framework centres on predicting an environmental variable from the full set of multivariate movement data. The measure of fit of this prediction is taken to be the metric that quantifies how much of the environmental variation relates to the multivariate variation in animal movement. We demonstrate the usefulness of this framework through a case study about the influence of grass availability and time since milking on cow movements using machine learning algorithms.

**Results:**

We show that on a one-hour timescale 37% of the variation in grass availability and 33% of time since milking influenced cow movements. Grass availability mostly influenced the cows’ neck movement during grazing, while time since milking mostly influenced the movement through the landscape and the shared variation of accelerometer and GPS data (e.g., activity patterns). Furthermore, this framework proved to be insensitive to spurious correlations between environmental variables in quantifying the influence on animal movement.

**Conclusions:**

Not only is our proposed framework well-suited to study the environmental influence on animal movement; we argue that it can also be applied in any field that uses multivariate biologging data, e.g., animal physiology, to study the relationships between animals and their environment.

**Supplementary information:**

**Supplementary information** accompanies this paper at 10.1186/s40462-020-00228-4.

## Background

Analysing animal movement is fundamental to ecology, because movement is arguably the most important way for animals to respond to their environment [1]. Quantifying the environmental influence on animal movement is therefore an important practice in ecology [2, 3]. As animal movement is inherently multifaceted, with aspects related to the movement of the animal through the landscape and aspects related to the movement of body parts, the movement process cannot be described with simplified descriptors without loss of information. On the contrary, a plethora of emergent patterns can be identified through these multifaceted movement descriptors, e.g., activity types (such as walking, foraging or resting) and collective movement properties [4, 5]. Technological advancements in the field of biologging currently allow for data on animal movement to be acquired at finer temporal and spatial scales and in increasing volumes, e.g., data on animal movement speed, movement path tortuosity, tri-axial acceleration of body parts, and heart rate patterns can now relatively easily be acquired [6–8]. These technological advancements provide opportunities to increase ecological understanding by analysing the full multivariate complexity of animal movement [7, 8]. This multivariate complexity of movement is not fully used in recent studies to infer the environmental influence on animal movement. Instead, quantifying the environmental influence on animal movement is currently often done through relating simplified movement descriptors, e.g., animal distributions, net displacements, diffusion rates, or distributions of step lengths and turning angles, to a suite of environmental variables, e.g., through canonical analyses, linear mixed models, semivariance approaches, diffusion approximations, step-selection functions, hidden Markov models, or state-space models [2, 3, 9–13]. Many of these approaches were not designed specifically for animal movement data, but are approaches that function generally well in quantifying the relationship of independent variables with one or several dependent variables. Although the simplification of movement descriptors prior to analyses is a useful practice to acquire ecological understanding, it almost necessarily leads to a reduction in the quantified environmental influence on multivariate animal movement as this influence may not become fully apparent in the simplified movement descriptors. Even more so considering that there are often multiple behavioural phenotypes that individuals of the same species can produce in a given set of environments [14], which can also be influenced by different internal states between individuals [1]. This consequently challenges the way that the analysis should be approached, as a multivariate analytic framework is required to quantify the overall influence of environmental variables on fine-scale multivariate animal movement data.

Data-driven machine learning methods provide a toolset to be able to model multivariate animal movement data and have been adopted by many animal ecologists over the past years [15, 16]. These machine learning methods have been used to automatically detect and classify animal species in images [17], to track moving animals in videos [18], to follow animal body postures and track body parts in videos [15], to flag when animals become sick using animal-mounted biologging sensors and videos [19], and to classify animal activities from biologging sensors [16]. Although machine learning has proven to be useful for movement ecology, it is often only used as a tool to transform raw data (e.g., images, videos, accelerometer readings) into informative data (e.g., species labels, animal locations, animal activity labels) [15, 16]. After these informative data have been generated, ecologists often use more traditional statistical methods to relate these data to environmental variables [2, 3, 7]. Machine learning has certainly generated ecological understanding via this way, but we posit that machine learning can also be used to acquire ecological understanding by directly relating animal movement data to environmental variables.

Here we propose a machine learning-based analytic framework, based on existing methods, to quantify the overall influence of an environmental variable on multivariate animal movement. After introducing the general framework, we demonstrate the usefulness of this framework with a case study about the influence of grass availability, time since milking, and wind speed on cow movements. Apart from quantifying the degree of coupling between the environment and cow movements, this case study shows that applying this framework can yield ecological insights. Finally, we discuss possible usages and constraints of this analytic framework. We contend that this framework contributes to the toolbox of ecologists studying the relationship between the environment and animal movement, behaviour, and physiology.

## Methods

Our analytic framework quantifies the influence of an environmental variable on animal movement by utilizing the multivariate richness of movement data. Instead of building a model to predict a simplified animal movement descriptor from a set of environmental predictors, i.e., the route of causal inference, we turn this around and build a model to predict an environmental variable from a large number of animal movement variables. By using animal movement variables, the model of this framework predicts a perceived environmental variable by the animal [6, 20]. Although predicting an environmental variable from movement data is the goal of the model, it is an intermediate step of the framework in order to quantify environmental influence on animal movement. In this framework it is key to use as many informative movement variables as possible, which could be meaningful human-constructed ecological (e.g., variables related to multiple classified animal activities), mathematical and/or physical variables, or abstract variables from an automated (deep learning) feature extraction algorithm. When effort is made to extract as many informative variables as possible from the animal movement data, chances are maximized that most of the variation of the environmental variable under scrutiny that is present in the data is captured. Furthermore, instead of creating the model as the end product during the analysis, the environmental variable should be predicted on a separate test dataset as the final step of the analysis. This follows from a data-driven and machine learning philosophy, in which complex multivariate models can be built that are not overfitted and therefore generalize better to new datasets. When distinguishing the train and test dataset, the test set used in the prediction of the environmental variable needs to be from a different temporal range than the train set that is used in the model building phase, due to autocorrelation in animal movement data that can otherwise cause the model to overfit [21]. The range of values in the test set of environmental variables (whether or not these are under scrutiny) should be comparable to the range of values in the train set, to prevent incorrect extrapolation. After generating model predictions on the test set, the coefficient of determination (*R*^*2*^) quantifies the fit of this predicted environmental variable from animal movement data to the measured environmental variable on a known scale and can thus be considered a metric on how much of the variation in the environmental variable influenced animal movement in a multivariate fashion (see Additional file 1) [22]. The measure of fit of the null model (i.e., no environmental influence) should be chosen depending on the algorithm that is used, which is *R*^*2*^ = 0 for algorithms that are able to always predict the mean of the response variable (e.g., Support Vector Regression and Random Forest Regression), even when the input variables are white noise. The measure of fit of this null model will then form the baseline value for which there is a 0% environmental influence and an *R*^*2*^ of 1 can always be interpreted as 100% environmental influence. Of course *R*^*2*^ should only be used as the measure of fit when modelling a continuous environmental response variable. With a discrete environmental variable, a classification approach should be undertaken, which is outside the scope of this study. However, to compare the influence of different environmental variables with each other fairly, the same measure of fit should be used.

**Fig. 1:**
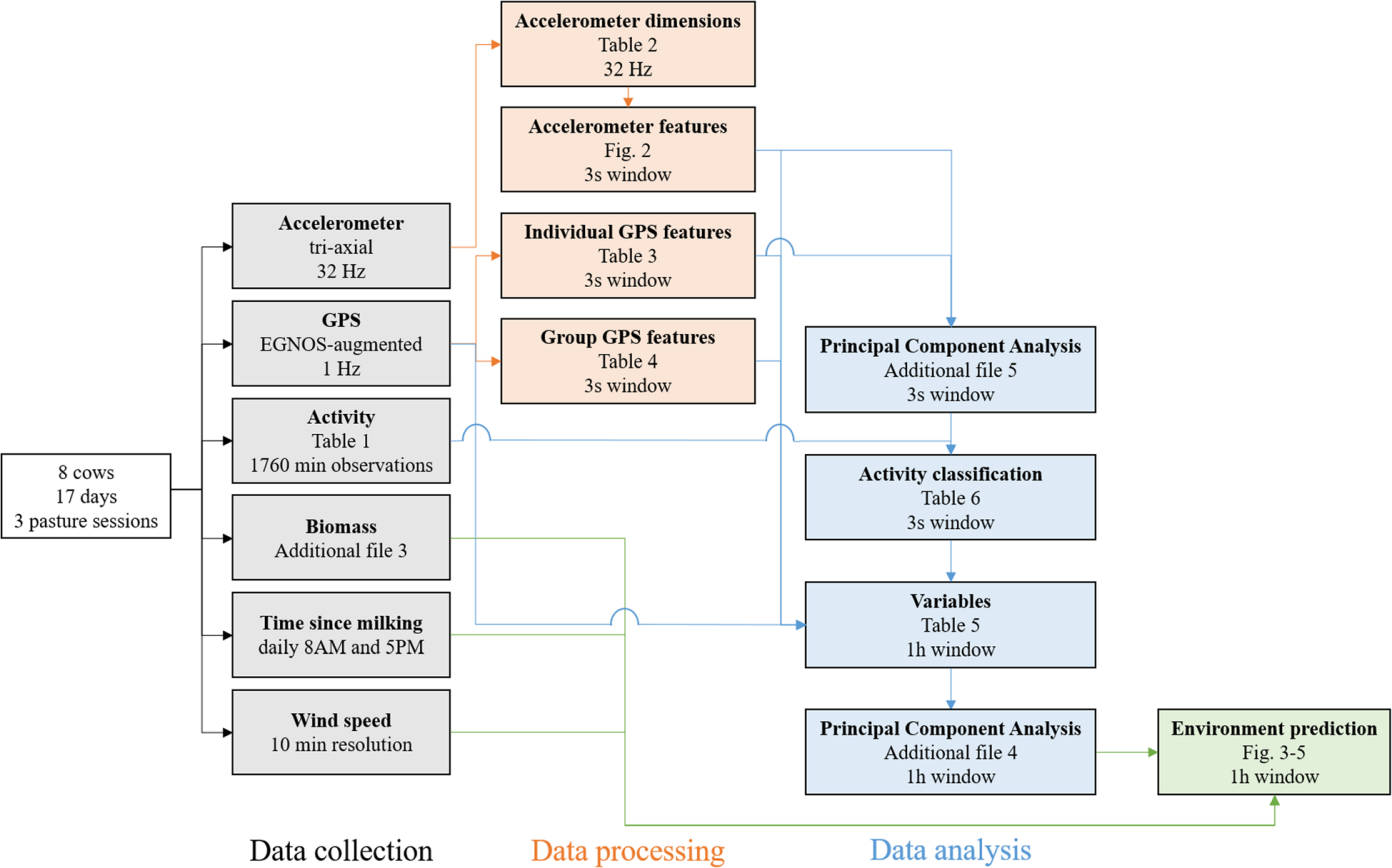
Flow chart of the summarized methodological approach for the case study

In order to demonstrate the usefulness of the proposed analytic framework, we applied this framework to a case study about the influence of resource availability (here grass biomass), time since milking, and wind speed on the movement of eight dairy cows in a pasture (Fig. 1). When animals are facing resource depletion, movement characteristics (through the landscape and of body parts), and emergent patterns like group (herd) characteristics, and time allocated to specific activities (e.g., foraging) often change, because animals need to invest more time and/or energy in searching for and acquiring resources [23, 24]. Cows in a pasture are a good model for such a case study, because this provides a relatively homogenous foraging arena. Time since milking is another variable that could substantially influence the movement of dairy cows, because it has been shown that the lactation stage of cows (a variable that is intuitively linked to time since milking regarding its effect on cow behaviour) influences the relative distribution of their activity patterns [25, 26]. Wind speed provided a good test case for our framework, because it was moderately correlated (*r* = 0.37) with grass biomass. We expected this correlation to be spurious and the effect of wind speed on cow movement to be negligible, because conditions were mild during the experiment (0–9 m s^− 1^).

The exact methodological approach that we describe for this case study is one possible implementation of our proposed analytical framework (Fig. 1). However, there are numerous possible implementations of this framework for other studies, which may be influenced by the problem statement, experimental setup, animal movement sensors, environmental data types, data quantity, etc. However, the property that all implementations should have in common is that the environmental influence on animal movement is quantified by predicting environmental variables from movement descriptors in a data-driven (viz., machine learning) approach, which uses the coefficient of determination as a measure to quantify this influence. This framework is fully based on existing machine learning methods that are already widely used in movement ecology [15, 16]. For example, the classification of animal activities from biologging data are often performed in a similar way, where movement features are extracted from the data and linked to known (supervised) or unknown (unsupervised) output values via a data-driven algorithm [4, 16, 27, 28]. In this framework we apply the same principle, but in a different setting, to predict the environment from multivariate animal movement. Furthermore, the interpretation of the coefficient of determination is atypical as well, where this measure is often used in movement ecology as solely a measure of model fit without an ecological meaning.

### Data collection

For this case study eight adult female Holstein-Friesian dairy cows were kept in controlled pastures that were small enough so that foraging lead to resource depletion over the course of several days. The experiment ran from 25 April until 11 May 2017. During the experiment, the cows’ movements were recorded continuously with e-Track neck collars (Noldus InnovationWorks, Wageningen, Netherlands), containing an EGNOS-augmented GPS receiver and a tri-axial accelerometer sensor. The cows were continuously kept on pasture at Carus animal facility in Wageningen, Netherlands (51°59′8″ N, 5°39′11″ E), and could move freely around as a single group during the experiment. Over the course of this period, we relocated the cows between three 0.32 ha pasture plots (sequentially five, six and six consecutive days in each plot). At every pasture switch the cows were housed inside the Carus facility for one night where they were offered fodder, so that they were not hungry at the start of a new pasture plot session. Furthermore, the cows were taken inside for milking and feeding every morning between 7:30 and 8:30 CEST and solely for milking every afternoon between 16:30 and 17:00 CEST. The time the cows spent on pasture was short enough to assume that the pasture did not increase in grass quality because of re-growth after grazing and only decreased in grass availability [29]. The short duration of the pasture sessions (approximately 1 day longer than when a commercial farmer would have moved the cows, as judged by the farm manager) ensured that the cows were not hungry, but only had to put more effort into foraging when time progressed. Furthermore, the collaring process did not put the cows under noticeable stress, more so because they were accustomed to continuously wearing a neck collar.

The sensors in the cows’ neck collars recorded GPS and accelerometer data during the experiment. The data were stored with a millisecond-accurate timestamp on a local SD memory card, which was replaced every 1 to 5 days together with the battery. GPS data were stored on the SD card with a 1 s interval. The accelerometer data were sampled with a variable frequency of 25–500 Hz, which were later down-sampled and linearly interpolated to a constant 32 Hz signal. Both the GPS and the accelerometer did not record data during some hardcoded multi-hour periods of inactivity, which were variable in duration and time of day, to save battery power. However, the time between GPS fixes was exactly 1 s in more than 99% of the cases. The precision of the GPS fixes was high, with 98% of the fixes having a Horizontal Dilution of Precision (HDOP) of less than two (a dimensionless unit; two is considered “excellent” precision). All GPS fixes with an HDOP of more than five, which were 0.5% of all fixes, were considered to be untrustworthy and filtered out of the final dataset. We also tested the accelerometer data for precision by placing the sensor on a stable, non-moving surface while it recorded for several minutes. The fluctuations in the recorded signal of all three accelerometer axes were small, 0.06 m s^− 2^ between the lowest and highest value, and were considered negligible and thus ignored.

Activity (or behaviour) observations were conducted on work days from 25 April to 9 May 2017. A single person visually classified the activities using focal-animal sampling with a pre-defined ethogram (Table 1). All activity types in the ethogram (grazing, walking, standing, standing while ruminating, lying, lying while ruminating) were mutually exclusive. Each individual cow was observed continuously for 10 min in the morning (10:00–13:00 CEST) and 10 min in the afternoon (13:00–17:00 CEST), in random order, resulting in a total observation time of 1760 min. During the observations, the start and end times of each displayed activity type from the ethogram were recorded. We conducted these observations to acquire annotations for an activity classification model. Representative acceleration plots of the three axes for the different activity types are provided (see Additional file 2).

**Table 1:** Ethogram. Descriptions of the recorded, mutually exclusive activity types

Activity	Description
Grazing	Foraging behaviour by chewing grass from the pasture whilst standing still or slowly moving with the head down
Walking	Taking at least two steps without grazing, either with the head up or down
Standing without ruminating	Standing on all four legs with head erect, without swinging its head from side to side and without ruminating
Lying down without ruminating	All four legs tucked underneath the torso or lying down on one side of its body without ruminating
Ruminating while standing	Masticating regurgitated feed, swallowing masticated feed or regurgitating feed while standing with head erect
Ruminating while lying down	Masticating regurgitated feed, swallowing masticated feed or regurgitating feed while lying down

We measured resource availability as dry matter grass biomass in kilograms per hectare, excluding stubble biomass. We determined time-varying biomass levels using a combination of field-measured biomass levels at specific time points, satellite-based biomass estimates derived from the Normalized Difference Vegetation Index (NDVI), and modelling of grass dynamics (see Additional file 3). Wind speed (m s^− 1^, mean speed 10 m above ground) were recorded at 10 min resolution during the experiments with a weather station on a grass pasture at the Veenkampen, Wageningen, Netherlands. This weather station is located one kilometre west of the pasture plots used for the experiments.

### Data processing

**Fig. 2:**
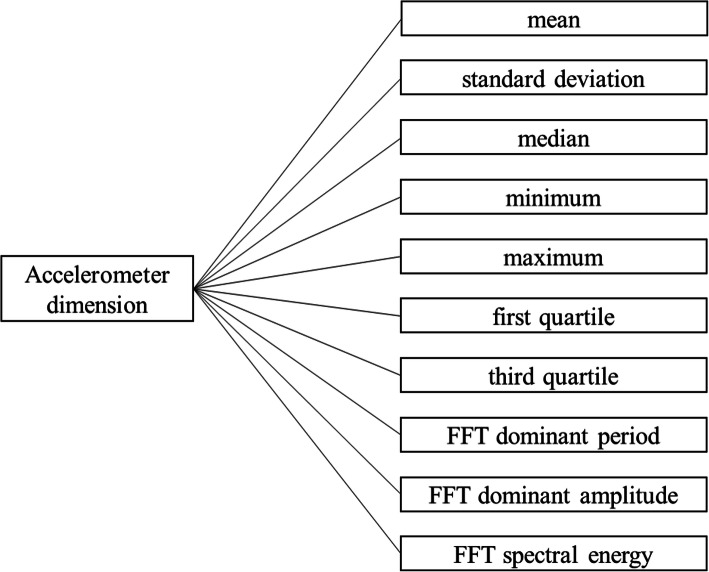
Statistics calculated per time window, cow and accelerometer dimension. FFT stands for Fast Fourier Transform

We used the pre-processed 32 Hz, tri-axial accelerometer signal as input for the accelerometer feature extraction. First, we converted all the records in the three-dimensional accelerometer dataset to 21 dimensions using multiple geometric transformations, i.e., resultant vectors, angles, solid angles, volumes and areas (Table 2). These dimensions constitute all geometric transformations of angles and distances in one, two and three dimensions. Considering that tri-axial accelerometer readings describe the movement forces in three dimensions, geometric transformations make sense from a physics perspective. More transformations could be considered, but these may lack to provide additional information to the feature set. Second, we divided the resulting dataset into non-overlapping time windows. We tried all window sizes in the range of 1 until 30 s and optimized this window size as a hyperparameter regarding the activity classification performance, where 3 s turned out to be the optimal window size (see Additional file 5). For every time window we computed multiple statistics per accelerometer dimension per cow, e.g., mean, standard deviation, quantiles and Fast Discrete Fourier Transform (FFT) parameters (Fig. 2). These statistics were chosen to provide summary statistics about both the time-invariant and sequential aspects of the data, given that accelerometer data also includes patterns in the frequency domain regarding animal activity (e.g., head movement of cows during grazing has a strong cyclic behaviour). We computed the FFT with the base R 3.6.2 stats package [30], of which we used the maximum FFT value as the dominant amplitude, the corresponding period of the dominant amplitude as the dominant period, and finally the sum of all squared FFT values as the spectral energy. Our list of computed statistics is not all-encompassing and more statistics can be thought of to describe patterns in the data, but these statistics are similar to the ones that are often used in activity classification with accelerometers [31, 32]. Furthermore, as these statistics were mainly used in the activity classification part of the analysis, we deemed the computed statistics sufficient when it resulted in a high performance during activity classification. Overall, computing all statistics for each dimension resulted in 210 accelerometer features per time window per cow.

**Table 2:** Dimensions extracted from the accelerometer data

Name	Formula	Description
*x*	*x*	raw accelerometer reading in the x axis
*y*	*y*	raw accelerometer reading in the y axis
*z*	*z*	raw accelerometer reading in the z axis
*r*_*xyz*_	$$ \left\Vert \begin{array}{c}x\\ {}y\\ {}z\end{array}\right\Vert $$	magnitude of resultant vector
*r*_*xy*_	$$ \left\Vert \begin{array}{c}x\\ {}y\end{array}\right\Vert $$	magnitude of resultant vector in x,y plane
*r*_*xz*_	$$ \left\Vert \begin{array}{c}x\\ {}z\end{array}\right\Vert $$	magnitude of resultant vector in x,z plane
*r*_*yz*_	$$ \left\Vert \begin{array}{c}y\\ {}z\end{array}\right\Vert $$	magnitude of resultant vector in y,z plane
*ϑ*_*xy*_	$\arctan \left(\frac{y}{x}\right)$	angle of resultant vector in x,y plane
*ϑ*_*xz*_	$\arctan \left(\frac{z}{x}\right)$	angle of resultant vector in x,z plane
*ϑ*_*yz*_	$\arctan \left(\frac{z}{y}\right)$	angle of resultant vector in y,z plane
*ϑ*_*z*_	$$ \arctan \left(\frac{z}{\left\Vert \begin{array}{c}x\\ {}y\end{array}\right\Vert}\right) $$	angle of resultant vector with x,y plane collapsed to 1 line
*ϑ*_*y*_	$$ \arctan \left(\frac{y}{\left\Vert \begin{array}{c}x\\ {}z\end{array}\right\Vert}\right) $$	angle of resultant vector with x,z plane collapsed to 1 line
*ϑ*_*x*_	$$ \arctan \left(\frac{x}{\left\Vert \begin{array}{c}y\\ {}z\end{array}\right\Vert}\right) $$	angle of resultant vector with y,z plane collapsed to 1 line
*Ω*_*x*_	$$ \arcsin \left(\frac{yz}{\left\Vert \begin{array}{c}x\\ {}y\\ {}0\end{array}\left\Vert \begin{array}{c}\\ {}\\ {}\end{array}\right\Vert \begin{array}{c}x\\ {}0\\ {}z\end{array}\right\Vert}\right) $$	solid angle of resultant pyramid base projected along x axis
*Ω*_*y*_	$$ \arcsin \left(\frac{xz}{\left\Vert \begin{array}{c}x\\ {}y\\ {}0\end{array}\left\Vert \begin{array}{c}\\ {}\\ {}\end{array}\right\Vert \begin{array}{c}0\\ {}y\\ {}z\end{array}\right\Vert}\right) $$	solid angle of resultant pyramid base projected along y axis
*Ω*_*z*_	$$ \arcsin \left(\frac{xy}{\left\Vert \begin{array}{c}x\\ {}0\\ {}z\end{array}\left\Vert \begin{array}{c}\\ {}\\ {}\end{array}\right\Vert \begin{array}{c}0\\ {}y\\ {}z\end{array}\right\Vert}\right) $$	solid angle of resultant pyramid base projected along z axis
*V*_*xyz*_	*xyz*	volume of resultant cuboid
*A*_*x*_	*yz*	area of resultant pyramid base projected along x axis
*A*_*y*_	*xz*	area of resultant pyramid base projected along y axis
*A*_*z*_	*xy*	area of resultant pyramid base projected along z axis
*A*_*xyz*_	$$ \frac{1}{2}\left\Vert \left[\begin{array}{c}x\\ {}y\\ {}0\end{array}\right]\times \left[\begin{array}{c}x\\ {}0\\ {}z\end{array}\right]\right\Vert $$	area of resultant triangle

We used the filtered 1 Hz GPS data as input for the GPS feature extraction. First, we transformed all the latitude, longitude coordinates to Cartesian coordinates by projecting them to zone 31 N of the UTM system (EPSG 32631). Second, we extracted a number of individual GPS features from the projected GPS coordinates per time window per cow, related to speed, turning angle, tangential velocity, mean squared displacement, and first passage time (Table 3), which are widely used metrics for path-level analyses in movement ecology [33]. The time windows were exactly the same as the time windows used in the extraction of the accelerometer features. Third, we extracted a number of group GPS features from the projected GPS data per time window per cow, related to group shape, group area, and distances and directions to other cows (Table 4), which are low-level geometric metrics similar to those used for 2D point clouds in computational geometry [34]. We determined which individual and group GPS features to compute by drawing fake GPS trajectories and animal clusters, after which we discussed which geometrical properties (e.g., tangential velocity: the linear speed of an animal moving along a circular path) could be extracted from these patterns. Furthermore, we computed ecological properties of animal trajectories that were known to us (e.g., Mean Squared Displacement: a measure of the deviation of the position of an animal with respect to a reference position over time) and searched the literature and animal movement related R packages for other ecological properties (e.g., First Passage Time: the time required for an animal to cross a circle with a given radius). We do not suggest that the provided list of computed features is all-encompassing, but we do suggest that spending time and effort in the engineering of features (or optimizing the architecture of a neural network in a deep learning approach) is an important part of our suggested framework. The more informative variation that is extracted from the raw data, the better the model could potentially perform and thus the better the quantified environmental influence on animal movement matches reality. Overall, computing both the individual and group GPS features resulted in 38 GPS features per time window per cow.

**Table 3:** Individual GPS features extracted per time window and cow

Dimension	Statistic	Description
Distance	Net gross ratio	Distance between first and last position divided by sum of distances of all segments
Speed	Mean	
	Standard deviation	
	Median	
	Minimum	
	Maximum	
	First quartile	
	Third quartile	
	Autocorrelation function index	Autocorrelation value at a lag of 1 s
	Brownian motion scaling parameter	See Eq. 1
Turning angle	ρ	Length of the mean resultant vector
	Autocorrelation function index of the absolute turning angles	Autocorrelation value at a lag of 1 s
Absolute tangential velocity	Mean	
	Standard deviation	
	Median	
	Minimum	
	Maximum	
	First quartile	
	Third quartile	
	Autocorrelation function index	Autocorrelation value at a lag of 1 s
Mean Squared Displacement	Diffusion coefficient	The value of *a* in the fitted model *MSD* = *aτ*^*b*^ on MSD values for τ from 1 to 6
	Diffusion power coefficient	The value of *b* in the fitted model *MSD* = *aτ*^*b*^ on MSD values for τ from 1 to 6
First Passage Time	Mean, 5 m radius	
	Variance of log, 5 m radius	
	Autocorrelation function index, 5 m radius	Autocorrelation value at a lag of 1 s
	Radius with maximum variance of log (integers from 1 to 10 m)	
	Linear regression coefficient log radius vs. log mean FPT

**Table 4:** Group GPS features extracted per time window and cow

Dimension	Statistic	Description
Net distances to other cows	Mean	
	Median	
	Minimum	
	# cows within 2 m radius	
	# cows within 4 m radius	
	# cows within 8 m radius	
	# cows within 16 m radius	
All mean cow coordinates	Group elongation index, φ	Variance explained by the first principal component through the mean x and y coordinates of all cows. Value lies by definition between 0.5 (when completely non-elongated, e.g., an exact circle) and 1 (when all coordinates lie on a straight line). Afterwards scaled between 0 and 1, by subtracting 0.5 and multiplying by 2.
	Group area proxy	*πσ*^2^(1 − *φ*); where *σ* is the standard deviation of the first principal component values. This measure assumes that the area can be estimated by considering the group as an ellipse. When completely non-elongated the area is *πσ*^2^ (where the variance *σ*^2^ is a proxy for the extent of the direction of elongation) and when fully elongated the area is 0.
Directions to other cows	ρ	Length of the mean resultant vector
	Periphery index	Maximum difference between consecutive directions, minus $\frac{2\pi }{\# cows-1}$ and divided by 2*π*

1 $$ \mathrm{Brownian}\ \mathrm{motion}\ \mathrm{scaling}\ \mathrm{parameter}=\sqrt{\frac{\sum \left(\left(\frac{\left[\begin{array}{ccc}\left(\frac{\Delta  x\left(t=1\right)}{\sqrt{\Delta  t}}-\frac{\sum \frac{\Delta  x}{\Delta  t}}{n}\right)& \cdots & \left(\frac{\Delta  x\left(t=n\right)}{\sqrt{\Delta  t}}-\frac{\sum \frac{\Delta  x}{\Delta  t}}{n}\right)\\ {}\left(\frac{\Delta  y\left(t=1\right)}{\sqrt{\Delta  t}}-\frac{\sum \frac{\Delta  y}{\Delta  t}}{n}\right)& \cdots & \left(\frac{\Delta  y\left(t=n\right)}{\sqrt{\Delta  t}}-\frac{\sum \frac{\Delta  y}{\Delta  t}}{n}\right)\end{array}\right]\left[\begin{array}{cc}\left(\frac{\Delta  x\left(t=1\right)}{\sqrt{\Delta  t}}-\frac{\sum \frac{\Delta  x}{\Delta  t}}{n}\right)& \left(\frac{\Delta  y\left(t=1\right)}{\sqrt{\Delta  t}}-\frac{\sum \frac{\Delta  y}{\Delta  t}}{n}\right)\\ {}\vdots & \vdots \\ {}\left(\frac{\Delta  x\left(t=n\right)}{\sqrt{\Delta  t}}-\frac{\sum \frac{\Delta  x}{\Delta  t}}{n}\right)& \left(\frac{\Delta  y\left(t=n\right)}{\sqrt{\Delta  t}}-\frac{\sum \frac{\Delta  y}{\Delta  t}}{n}\right)\end{array}\right]}{n}\right)\left[\begin{array}{cc}1& 0\\ {}0& 1\end{array}\right]\right)}{2}} $$;

in which *n* is the number of records.

### Data analysis

We used the accelerometer features and individual GPS features per time window per cow for which activity observations were undertaken as input data for the activity classification models (Fig. 2, Table 3), which we first converted to principal components. We linked the time-matched activity observations to these input data and used the activity type as output variable for the classification models. We trained a multi-class classification model for the activity types: grazing, walking, standing and lying down. As a second step after the main activity classification we also trained a binary classification model for ruminating, with an extra input variable that indicated standing versus lying down. We tried for both classification models a Support Vector Machine (SVM) with a Radial Basis Function (RBF) kernel and a one-against-one approach, implemented in the e1071 package for R 3.6.2 [30, 35], and a Random Forest (RF) with 500 trees, implemented in the randomForest package [36]. To prevent overfitting due to autocorrelation in the data we randomly assigned each hour of the dataset into a train (80%) or test set (20%) and performed 5-fold cross-validation on the train set, which was also split per hour at each of the 5 cross-validation iterations [21]. To find the optimal hyperparameters for the models (number of principal components and time window size for both SVM and RF; *cost*, *gamma* and class weights for SVM; and *mtry*, *sample size* and *node size* for RF), we used an extensive grid search on a High Performance Cluster of Wageningen University, Netherlands (see Additional file 5). We started the grid search with a coarse resolution search that covered a large range of all hyperparameters, to make sure that the global optimum was covered and to get a feel for the performance landscape. We zoomed in with a finer resolution during a second grid search and finished with an even more zoomed in and finer resolution during a final grid search. We determined the optimal classification model and hyperparameters by selecting for the highest mean balanced accuracy during cross-validation (Eq. 2). The classification models with the highest performance during cross-validation were then evaluated for performance on the test dataset. Finally, we used the models to predict the displayed activity type (grazing, walking, standing or lying down) and whether or not the cows were ruminating, for all the time windows and cows with available sensor data.

2 $$ \mathrm{mean}\ \mathrm{balanced}\ \mathrm{accuracy}=\frac{\sum \limits_{x=1}^n\left(\frac{1}{2}\left(\frac{TP_x}{P_x}+\frac{TN_x}{N_x}\right)\right)}{n} $$

where *x* is a class; *n* is the number of classes; *TP* is the number of true positives; *P* is the number of positives; *TN* is the number of true negatives; and *N* is the number of negatives.

We computed the dataset for the environmental variable predictions per cow over one-hour time windows. The window size that is chosen has of course an influence on the results, as the effect of an environmental variable on animal movement data varies with temporal scales [37]. In short, the window size that is chosen represents the scale at which the animals’ behavioural decisions are made [37]. The choice of this temporal scale should therefore be chosen in line with the study’s aim and based upon ecological considerations, which are different for every study. We chose a window size of 1 h for a combination of two reasons: 1) it makes sense from an ecological point of view, as the considered environmental variables likely influence cow behaviour on this temporal scale, and 2) because it traded off the number of resulting data records (number of rows in the dataset after applying the 1 h window) and the convergence of variables well; meaning that the resulting dataset consists of hundreds of records (thereby being enough for a data-driven machine learning approach) and each record was based on 1200 (1 h divided by 3 s) underlying records or more (thereby making sure that the inherent heterogeneity of animal movement is taken into account by averaging it out over a large enough period). The calculated variables consisted of multiple variable sets, based on the source of the data (GPS or accelerometer), organizational level (group or individual), transformation type, and variables conditional on foraging (Table 5). We did not consider variables conditional on other activity types than foraging, because the cows sometimes did not display one of the other activity types during a 1 h time window. This resulted in a total of 548 variables per cow per one-hour time window. We standardized these variables (to zero mean and unit variance) per combination of day/night and cow ID to account for differences in nocturnal and diurnal activities of cows and individual differences in movement characteristics, group characteristics, and activities. These standardized variables were used as input for a principal component analysis, but were first one by one visually checked for symmetric unimodality by inspecting the histograms and normal Q-Q plots. Two of the 548 variables displayed signs of bimodality and eight variables appeared to be somewhat heavy-tailed. Due to the low number of variables that showed these deviations and due to the small severity of these deviations, we decided not to correct these ten variables and thus left all standardized variables untransformed. Moreover, symmetric unimodality is not an actual requirement of a principal component analysis, but it does result in a better centring and scaling of the principal components. After that we converted the standardized variables to principal components separately for the GPS and accelerometer variables and linked these principal components to the mean grass biomass, time since milking, and wind speed values per hour (see Additional file 4). To prevent overfitting of the model due to autocorrelation of the time series, we trained the model on the data of all cows from two of the three pasture plot sessions (*n* = 600, viz., number of rows in the train set) and tested the model on the data of all cows from the other pasture plot session (*n* = 259, viz., number of rows in the test set). We used the second pasture plot session as our test set, because its range of biomass values fell within the range of biomass values of the first and third pasture plot session.

**Table 5:** Calculated variable sets per cow over one-hour time windows

Variable set	Statistic	Transformed data
Individual GPS	All statistics from Table 3	1 Hz GPS data
Proportion activity	Proportion	Predicted activity per three-seconds window (Table 1)
Individual GPS distribution parameters while grazing	Mean and standard deviation of log-transformed data	Median speed and median absolute tangential velocity per three-seconds window while grazing (Table 3)
Median group GPS	Median	Group GPS features per three-seconds window (Table 4)
SD group GPS	Standard deviation	Group GPS features per three-seconds window (Table 4)
Median individual GPS while grazing	Median	Individual GPS features per three-seconds window while grazing (Table 3)
SD individual GPS while grazing	Standard deviation	Individual GPS features per three-seconds window while grazing (Table 3)
Median group GPS while grazing	Median	Group GPS features per three-seconds window while grazing (Table 4)
SD group GPS while grazing	Standard deviation	Group GPS features per three-seconds window while grazing (Table 4)
Median accelerometer while grazing	Median	Accelerometer features per three-seconds window while grazing (Fig. 2)
SD accelerometer while grazing	Standard deviation	Accelerometer features per three-seconds window while grazing (Fig. 2)

To predict the environmental variables we built a Support Vector Regression (SVR) model with a RBF kernel and a Random Forest Regression (RFR) with 1000 trees on the train set with both GPS and accelerometer principal components, with only GPS components, and with only accelerometer components. These models are time-invariant, as they assume independence between the data records, and are particularly well-suited to model complex interactions between a large number of variables. To find the optimal hyperparameters for the models (number of principal components for both SVR and RFR; and *cost*, *gamma* and *epsilon* for SVR), we used a grid search (following the same procedure as during the grid search of the activity classification) on a High Performance Cluster of Wageningen University, Netherlands (see Additional file 4). We did not optimize any other RFR hyperparameter, because the performance improved barely compared to the default values during a trial analysis. We determined the optimal hyperparameters by selecting for the highest *R*^*2*^ on the test set (Eq. 3). Ideally, (cross-)validation is performed before a test set evaluation to prevent overfitting in hyperparameter space, but the limited quantity of data records in our case study prevented us from setting aside more data from the train set. However, we prevented overfitting in hyperparameter space by not optimizing the hyperparameters of the RFR and by limiting the amount of hyperparameter values that were tested for the SVR.

3 $$ {R}^2=1-\frac{\sum_i{\left({y}_i-{f}_i\right)}^2}{\sum_i{\left({y}_i-\overline{y}\right)}^2} $$

where *y* is the vector of actual values and *f* is the vector of predicted values.

## Results

**Fig. 3:**
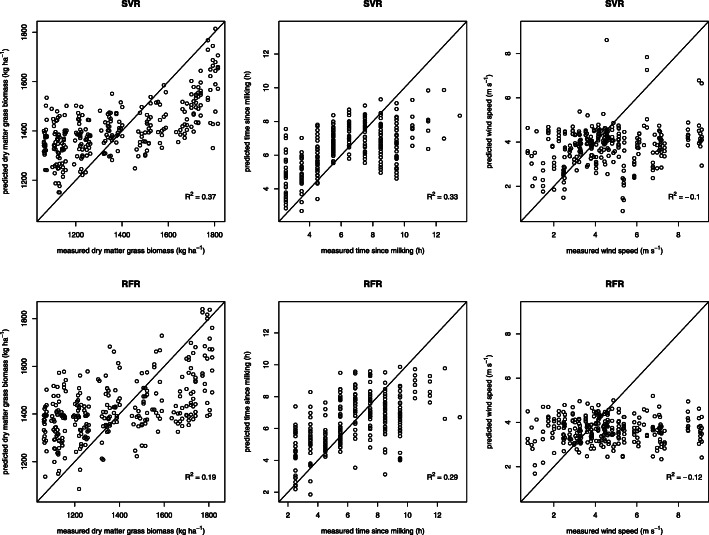
Left to right: measured versus predicted grass biomass, time since milking and wind speed using GPS and accelerometer data. Top: Support Vector Regression predictions. Bottom: Random Forest Regression predictions

**Fig. 4:**
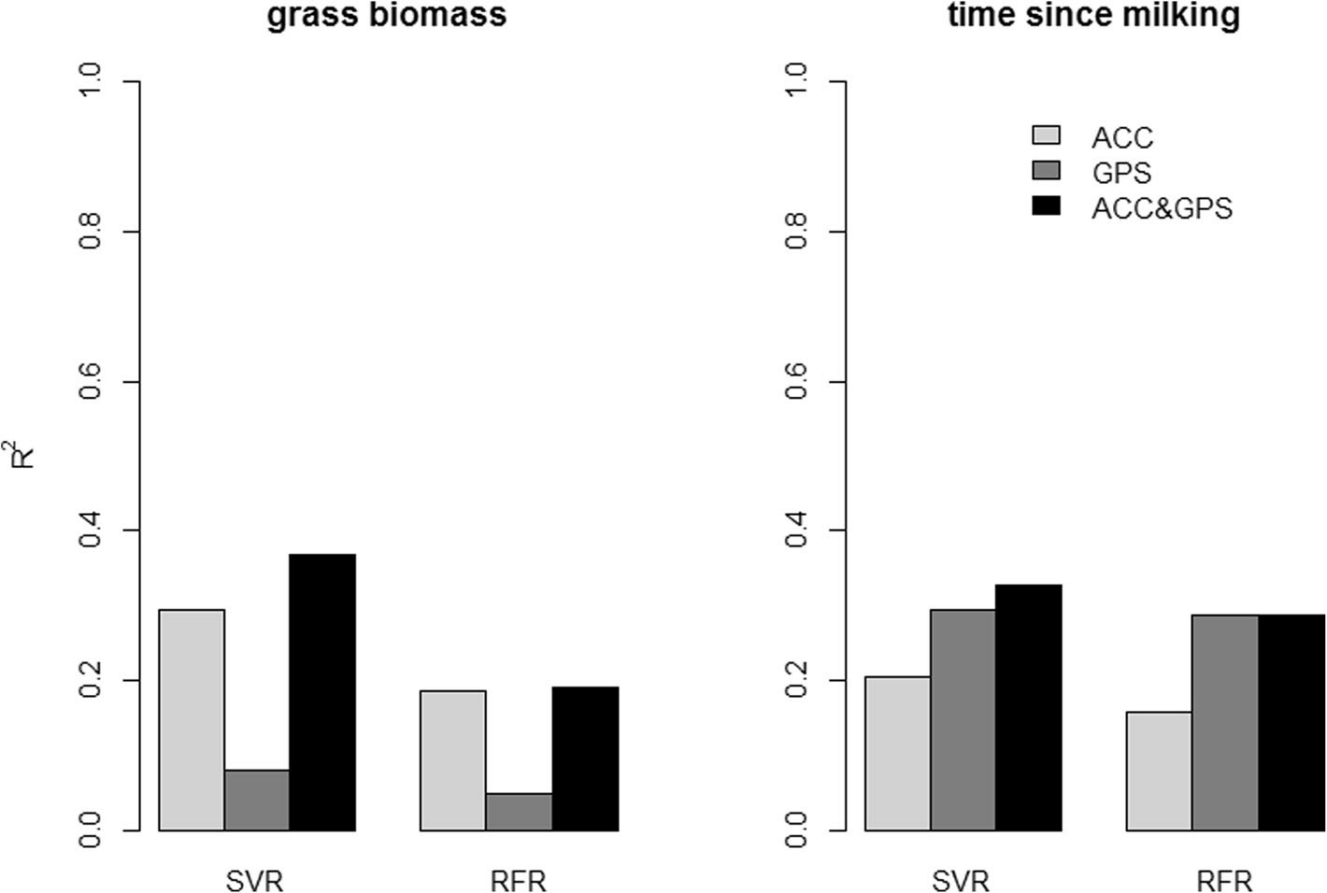
Explained grass biomass and time since milking variation using Support Vector Regression models (SVR) and Random Forest Regression Models (RFR) with a GPS, accelerometer (ACC) and combined dataset

**Fig. 5:**
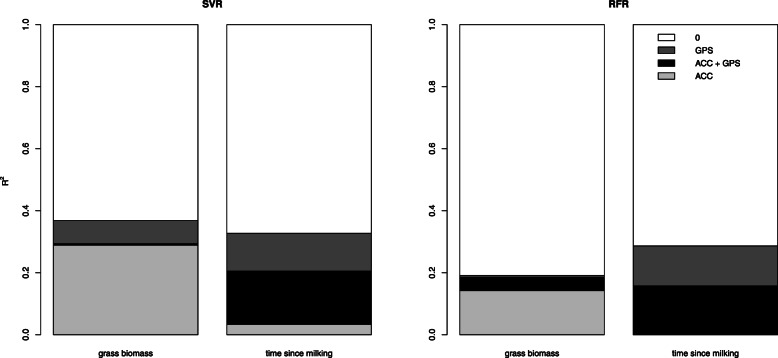
Variation partitioning of accelerometer (ACC) and GPS data with Support Vector Regression models (SVR) and Random Forest Regression models (RFR) for grass biomass and time since milking

The general cline of grass biomass is predicted by both models, but the steepness is not entirely captured (Fig. 3). The time since milking cline is quite accurately matched from 0.5 to 6.5 h, but after 6.5 h it levels off for both models (Fig. 3). For wind speed both models were not able to make accurate predictions (Fig. 3). Overall the SVR models outperformed RFR in predicting the environmental variables from cow movement data (see Additional file 4). When analysing the explained variation of the models with only accelerometer or GPS datasets, the qualitative differences between the explained variation of the different response variables for both algorithms are comparable (Fig. 4). However, SVRs are apparently better capable of using the interaction between variables in the mixed-sensor dataset to increase the explained variation, while RFRs are hardly able to do so with our data (Figs. 4 and 5). Both models indicate that grass biomass influences accelerometer data substantially more than GPS data, while the reverse is true for time since milking (Figs. 4 and 5). Furthermore, for time since milking the explained variation by accelerometer data is largely shared with GPS data (Fig. 5). Finally, the optimization of the hyperparameters was also done on datasets of each cow separately, which resulted into approximately the same hyperparameters and performance when compared to the model for all cows combined. Therefore, we concluded that cows responded to changes in resource availability and time since milking in approximately the same manner and we thus decided to use the models for all cows combined.

Some of the variables used in our model were based on the automated activity classifications of the cows’ sensor data and visual observations. SVMs outperformed RFs for all these activity classification tasks with our data, so we only used the predictions of the SVMs. The best performing SVM classification model of the main activity types achieved 91.7% mean balanced accuracy on the test set and the best performing SVM model of rumination 90.9% (see Additional file 5). While we maximized the mean balanced accuracy during cross-validation, also kappa, Matthews Correlation Coefficient, mean *F*_*1*_ and mean True Skill Statistic were maximized at the same time (Table 6). Moreover, the confusion matrices of both models show that, in addition to a high accuracy, the relative frequency of misclassification of each activity type was approximately equal (see Additional file 6). This means that the models were not overclassifying a specific activity type over another. Furthermore, we have found no substantial inter- or intra-cow activity classification performance differences. We thus considered the SVM activity classification models good enough to reliable predict the activity types based on the movement sensor data, even more so because the classification performance was higher or comparable to other cow activity classification studies [4, 27, 28].

**Table 6:** Performance measures on the test set of the best performing SVM activity classification models (*g = grazing; w = walking; s = standing; l = lying*) [38]

	Main activity types	Rumination
*Balanced accuracy*	μ = 91.7% (*g = 94.2%; w = 84.5%; s = 90.2%; l = 97.9%*)	90.9%
*Accuracy*	94.2%	90.9%
*Kappa*	88.0%	79.8%
*Matthews Correlation Coefficient*	88.0%	80.0%
*True Skill Statistic*	μ = 83.4% (*g = 88.3%; w = 69.0%; s = 80.4%; l = 95.8%*)	81.8%
*F*_*1*_	μ = 88.0% (*g = 96.6%; w = 76.6%; s = 83.4%; l = 95.6%*)	86.6%
*Precision*	μ = 90.0% (*g = 95.9%; w = 84.9%; s = 85.1%; l = 94.2%*)	82.8%
*Recall*	μ = 86.5% (*g = 97.3%; w = 69.8%; s = 81.7%; l = 97.0%*)	90.9%
*Negative predictive value*	μ = 97.4% (*g = 93.9%; w = 98.0%; s = 98.3%; l = 99.4%*)	95.4%
*True negative rate*	μ = 96.9% (*g = 91.0%; w = 99.2%; s = 98.6%; l = 98.8%*)	90.9%

## Discussion

In the case study we quantified (on a one-hour resolution) that 37% of the variation in resource availability influenced cow movements (consisting of movement through the landscape, body part movement, and emergent patterns like group characteristics, and displayed activities) and time since milking influenced it for 33%, while wind speed did not influence it noticeably (Fig. 3, 4 and 5). These results support our expectations that both resource availability and time since milking are important in shaping the movement of cows, but that wind speed (during relatively mild conditions) is not. Furthermore, it seems that the moderate correlation between resource availability and wind speed was indeed spurious. This framework proved to be insensitive to this spurious correlation, as it did quantify the influence of wind speed on cow movement to be 0%. Furthermore, the Support Vector Regression (SVR) models performed overall better than the Random Forest Regression (RFR), especially when confronted with a dataset with both GPS and accelerometer variables, but the qualitative patterns when comparing the three different environmental influences to single-sensor movement datasets were the same for both algorithms. Due to the SVRs higher performance, we do consider it to be the better alternative over RFR for this analytical framework when dealing with hyperdimensional datasets, especially when variables from multiple sensors are mixed. Moreover, we found that resource availability influenced accelerometer variables (29%) more than GPS variables (8%), but this influence on GPS variables still was largely independent from accelerometer variables (less than 1% of the total variation was shared). This indicates that, at this temporal scale and with these computed movement variables, the individual movement of cows through the landscape and the spatial group characteristics hardly contained any signature of resource availability and that almost all of the influence of resource availability on cow movements became apparent from the accelerometer variables of the cows’ neck during grazing. The accelerometer variables of the cows’ neck during grazing, being descriptive for bite frequency and bite force (Table 2, Fig. 2 and Table 5), probably link more explicitly to grazing behaviour than GPS variables do. These accelerometer variables are probably influenced more by resource availability than GPS variables, because grazing behaviour in cows is closely linked to resource availability [39]. The opposite was found for time since milking, which influenced GPS variables (29%) more than accelerometer variables (21%), with a lot of their explained variation being shared (17% of the total variation). This links well to our previous argument about that the accelerometer variables are shaped for a large part by the cows’ neck movement during grazing, which is intuitively more heavily influenced by grass biomass than by time since milking. Previous studies also found that the lactation stage, a variable that we expected to be linked to time since milking regarding its effect on cow behaviour, influences the relative distribution of cow activity patterns and cow movement through the landscape [25, 26]. This supports our finding about a higher influence on GPS variables with a large shared influence with accelerometer variables, because the movement through the landscape is measured by GPS variables and the activity patterns are measured by both GPS and accelerometer variables. Finally, the estimated model parameters were similar for all cows, indicating that the cows responded to changes in resource availability and time since milking in the same way. However, it should be noted that all the results that are presented above are of course context dependent. With a different experimental setup, e.g., indoor instead of pasture housing or different ranges of environmental variable values, the quantified influences can change. As is the case with nearly all modelling efforts, this framework is also only able to provide sensible results about the system for which data is available.

Our case study illustrates how the proposed analytic framework can quantify the influence of an ecological variable on animal movement. Having this quantification as the goal of the analytic framework, human interpretation and understanding of the correlative relationships within the model is initially of lesser importance. The goal is to build a model that can predict as much of the variation in the measured environmental variable as possible, by not limiting the model’s complexity to facilitate human interpretation. Only then the aim is to quantify the overall influence of the environmental variable on animal movement. This analysis could be followed by a stage where the researcher is selective in the choice of movement variables, to study which movement variables are mainly influenced by the environmental variable. Due to the way the framework is set up, the environmental influence on multivariate animal movement will by definition always be higher or equal to the environmental influence on a subset of the animal movement variables. Thus, using this framework to first determine the environmental influence on multivariate animal movement and afterwards determine the influence on specific subsets of movement variables, allows for an analysis that shows in which movement variables the environmental influence is most or least visible. This is demonstrated in our case study, where resource availability mainly influenced accelerometer variables and much less GPS variables, indicating that resource availability was more tightly linked to the cows’ movement of body parts than to their movement through the landscape. The opposite was true for time since milking, where also the explained variation by the accelerometer data was largely shared with GPS data. Furthermore, this framework allows for a comparison between the influences of multiple environmental variables to animal movement whilst being insensitive to moderate spurious correlations between environmental variables, which is also shown in our case study with regards to the influence of wind speed. Therefore, this framework could be well suited for exploratory analyses of the link between environment and animal movement.

In our framework the influence of the environment on animal movement is quantified, but the difference with previous studies (using low-dimensional movement descriptors [2, 3]) is that our result is quantified by how much of the variation in the environment can be predicted by observing the movement (instead of the other way around). Terming this quantified measure “environmental contribution”, it should be noted that the environmental contribution to animal movement (i.e., the variation in an environmental variable that is traceable in animal movement data) is not the same as the environmental dependency of animal movement (i.e., the variation in animal movement that is dependent on an environmental variable), where potentially the environmental contribution can be large but the dependency small or vice versa. To accommodate for a multivariate analysis of animal movement we determine environmental contribution instead of the easier interpretable environmental dependency. In movement ecology usually the environmental dependency of animal movement is the focus of analyses, as this allows for the determination of the direction and strength of the environmental influence on an animal movement variable. Therefore, post hoc analyses that link environmental variables to a simplified animal movement descriptor can supplement our proposed multivariate analytic framework in order to study the route of causal inference [2, 3].

**Fig. 6:**
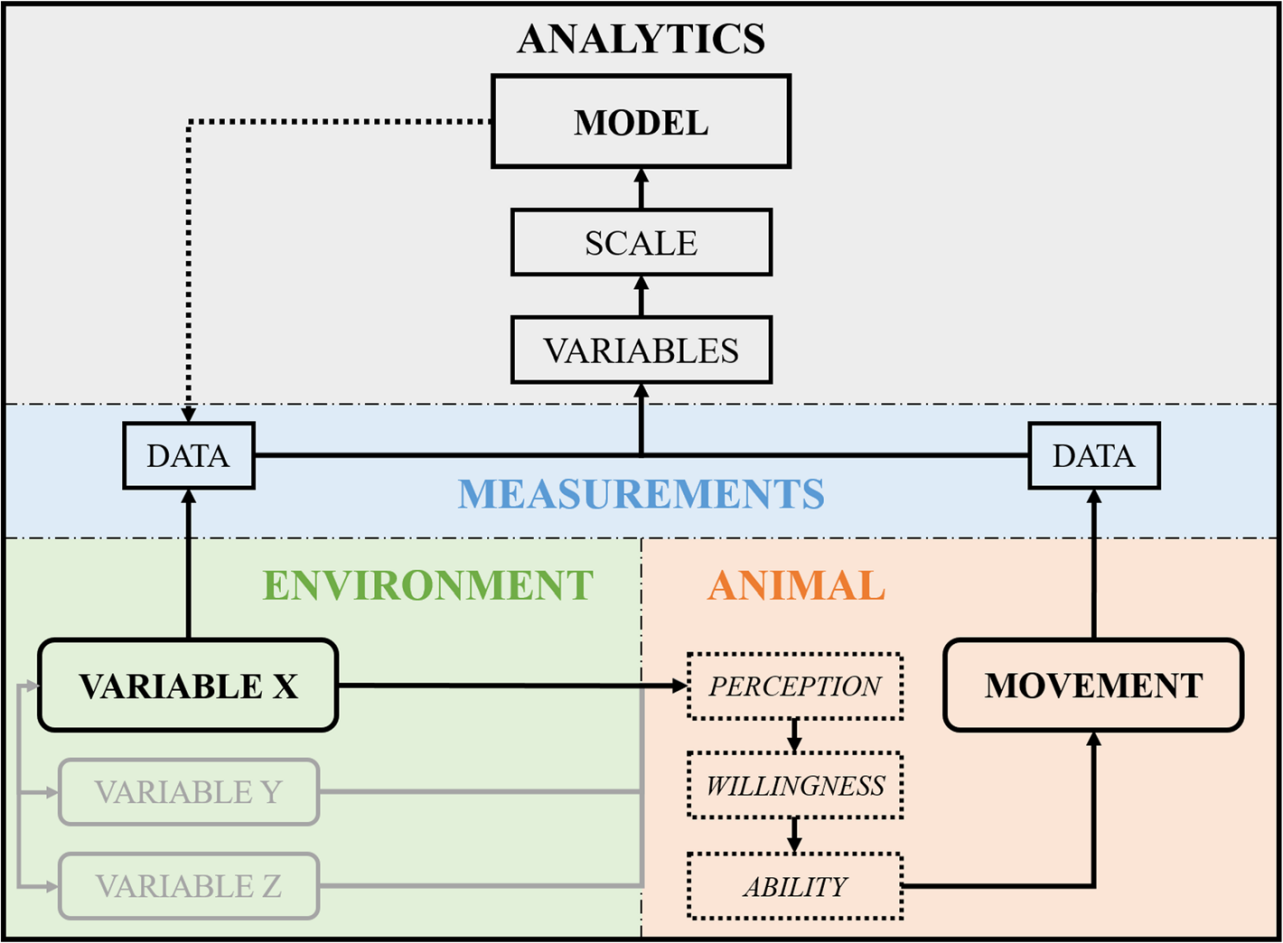
Conceptual model of the relationship between an environmental variable, animal movement and a predictive model to determine the influence of an environmental variable on multivariate animal movement. Dotted blocks are latent variables, rounded blocks are measurable variables, greyed out blocks are unmeasured variables, and straight blocks are known variables, values, or objects. The dotted arrow displays the predictive analysis following up on the model building phase

Various factors in the relationship between the environment and animal movement influence the quantification of the environmental influence on animal movement (Fig. 6). First, many environmental variables are correlated and interact with each other in their influence on the animal’s decision making and, thus, movement [1]. When the influence of a single environmental variable on animal movement is under scrutiny, these correlations and interactions with other environmental variables need to be taken into consideration. In the proposed analytic framework we do not distinguish between the independent, shared, and interaction influences of environmental variables on animal movement [9], which is different from the independent and shared influence on multiple subsets of the movement variables as described in our case study. As a consequence, both the direct and indirect influences of an environmental variable on animal movement are combined into a single metric. Future research could potentially be aimed at the distinction between these influence types of multiple environmental variables on multivariate animal movement, e.g., by using multi-target (Support Vector) regression and variation partitioning procedures [40, 41]. Furthermore, when the influence of an environmental variable on animal movement is quantified, it is important that the movement itself does not influence the environmental variable directly at that point in space and time as well. Social proximity is for example an important variable in the shaping of individual animal movement, but individual movement parameters also directly shape collective movement patterns [42]. The fit of a model with social proximity as response variable and individual movement variables as input data would then not be solely the influence of an environmental variable anymore. This could consequently yield unrealistically large values of the explained variance, which should be prevented.

In the relationship between the environment and animal movement, the animal’s internal state (*“why move?”*), motion capacity (*“how to move?”*), and navigation capacity (*“where to move?”*) are also involved [1]. The animal’s internal state is composed of many different factors, e.g., physiological “need” (hunger, fear, etc.), physical characteristics (age, sex, body condition, etc.), and personality differences (laziness, level of sociality, etc.), that combined result in a certain response by the animal when confronted with a set of environmental variables at certain moment in time [1]. We translate this combined net effect of the internal state factors into the willingness of the animal to respond to the environment (Fig. 6). The motion and navigation capacity can be translated into the ability of the animal to respond. Another factor that is involved, even before the animal can decide whether it is willing and able to respond, is the animal’s perception of the environment [20]. Only when an animal can observe changes or differences in an environmental variable can it decide to respond in a certain way. Because of the aforementioned latent variables – perception, willingness, and ability – the movement of the animal is not purely a deterministic function of a fixed set of environmental variables [1]. These latent variables can thus cause a partial environmental influence on animal movement. Furthermore, these latent variables are in part individual-specific [1], which is why differences between individuals should be taken into consideration by standardizing the movement variables per individual and/or adding individual identifiers as variables to the model.

Other factors, which are more data-related, also influence the quantification of the environmental influence on animal movement (Fig. 6). First, environment and animal movement are linked through sensor measurements, which influence the outcome of the analysis through varying sensor types, resolution, extent, and precision. Second, the movement variables that are computed from the animal movement data to describe the movement process determine how much of the environmental influence on animal movement is traceable in the data. Therefore it is key to extract as many informative movement variables from the animal movement data as possible in this proposed framework (or optimize the architecture of a neural network in a deep learning approach), because ideally all inherent variation needs to be extracted from the movement data to quantify the total environmental influence and to compare the influence of different environmental variables fairly. In our case study, the best performing models had a selected number of principal components with a relatively low cumulative proportion of variance, especially for the GPS variables (see Additional file 4), which suggests that enough variation had been extracted from the raw data to make a good prediction about the environmental influence on animal movement. Although the best performing model does not necessarily equate a good model, so it could theoretically also be that we missed to extract some extra informative variables from the raw data, which could otherwise have resulted in an even better performing model. Third, the temporal scale at which these variables are computed determine the temporal scale for which the influence of the environmental variable on animal movement is quantified. As the effect of an environmental variable on animal movement data varies with temporal scales, the choice of the temporal scale of the variables is relevant [37]. Finally, the algorithm that is used to predict an environmental variable from animal movement data influences the level of fit that can be attained, which is demonstrated in our case study with SVR outperforming RFR on all occasions. Algorithms that can model complex interactions between variables are often able to make better predictions of the response variable, e.g., RFR, SVR, and Neural Network Regression, likewise are algorithms that take into account the sequence of time series data, e.g., Recurrent Neural Network. Quantitative comparisons between the influences of different environmental variables on animal movement can thus only be done reliably when the same algorithm is used on the same underlying animal movement dataset.

Apart from only using the *R*^*2*^ of the model predictions to acquire ecological insights, the patterns of the observed vs. predicted plots can also potentially generate insight. For an environmental variable to influence animal movement, the animal’s perception, willingness, and ability are conditionalities (Fig. 6). Therefore, certain parts of the environmental variable’s range might be better predicted by the model than other parts. It could be argued that this could be an explanation for the better SVR predictions during intermediate grass biomass compared to low and high biomass levels, thereby creating a lower overall slope of the predictions compared to the observations (Fig. 3). However, apart from animal perception, willingness, and ability, other factors might also influence patterns of the observed vs. predicted plot (Fig. 6). In this case the algorithm might be the underlying cause for the lower overall slope of the SVR biomass predictions, due to a “regression toward the mean” characteristic (see Additional file 7). Furthermore, the overall gradient of the time since milking predictions follows the measurements quite accurately for both models from 0.5 to 6.5 h, but after 6.5 h it levels off (Fig. 3). This suggests that until 6.5 h cows continue to change their movement in response to the time since they were last milked, but after 6.5 h there is no noticeable change in movement anymore. Besides a potential behavioural ecological cause for this pattern, it could also be (partially) caused by correlations with other time variables due to our experimental setup where the cows were milked two times a day around the same time of day. Follow-up studies could focus on these predicted time since milking patterns, where the experimental setup should contain multiple groups of cows that are milked at different times of the day. Finally, apart from concluding that wind speed probably has no noticeable effect on cow movement in this study (Fig. 3), it becomes clear that the model performance suffered from some higher wind speed values in the test set compared to the train set (thereby generating an *R*^*2*^ lower than 0).

## Conclusions

We developed an analytical framework from existing methods that can quantify the environmental influence on animal movement while preserving the multifaceted nature of the movement process. Apart from providing a measure of the tightness of coupling between an environmental variable and animal movement, the prediction of an environmental variable from animal movement data can be a useful application in itself as the unique property of this predicted variable is that it represents the perceived environmental variable by the animals. This framework demonstrates that the possible applications of machine learning methods extend beyond the ability to transform raw into informative data to acquire ecological understanding, and that machine learning can also be used to directly relate movement data to environmental variables.

The applicability of our multivariate analytic framework extends beyond animal movement. With the recent increase in biologging practices, more and more variables of animal data are acquired [6–8]. These data do not only encompass animal movement, but for example also animal physiology, which can be related to environmental variables as well using the same framework as presented in this study [6–8], e.g., by relating heart rate patterns to terrain characteristics or physical fitness metrics to climate conditions. Apart from using this analytic framework to quantify environmental influence on animal biologging data, the computation of perceived environmental variables can allow researchers and managers to monitor the perceived habitat of animal species [6, 20]. This way, the habitat quality in natural areas, e.g., in terms of resources, can be assessed more precisely regarding the needs of specific, sensor-equipped, focal animals [6, 43]. Furthermore, with the results presented here, the management of pasture-fed cattle can be optimized by detecting the appropriate time to move cattle to a more resource-rich area or towards a milking machine, without measuring resource availability or milk content in the udder directly. Finally, we argue that our proposed data-driven analytic framework to quantify environmental influence on animal biologging data is a valuable tool for explorative and comparative analyses on the relationship between the environment and animal movement, behaviour, and physiology.

## Supplementary information

**Additional file 1.**

**Additional file 2.**

**Additional file 3.**

**Additional file 4.**

**Additional file 5.**

**Additional file 6.**

**Additional file 7.**

## Data Availability

Our code and data are available in the 4TU.ResearchData repository: 10.4121/uuid:e552fe57-ab4f-4e31-83e3-82e1cbc06a70 [44].

## Abbreviations

*R*^*2*^: Coefficient of Determination
HDOP: Horizontal Dilution of Precision
NDVI: Normalized Difference Vegetation Index
FFT: Fast Discrete Fourier Transform
MSD: Mean Squared Displacement
FPT: First Passage Time
SVM: Support Vector Machine
RBF: Radial Basis Function
RF: Random Forest
SVR: Support Vector Regression
RFR: Random Forest Regression
ACC: Accelerometer
