# Supplementary material for: Inferring an animal’s environment through biologging: quantifying the environmental influence on animal movement
Source: Mov Ecol. 2020 Oct 19;8:40. doi: 10.1186/s40462-020-00228-4 (PMC7574229; doi:10.1186/s40462-020-00228-4)
Supplement: Supplementary file 1 — Additional file 1. [file 40462_2020_228_MOESM1_ESM.docx]

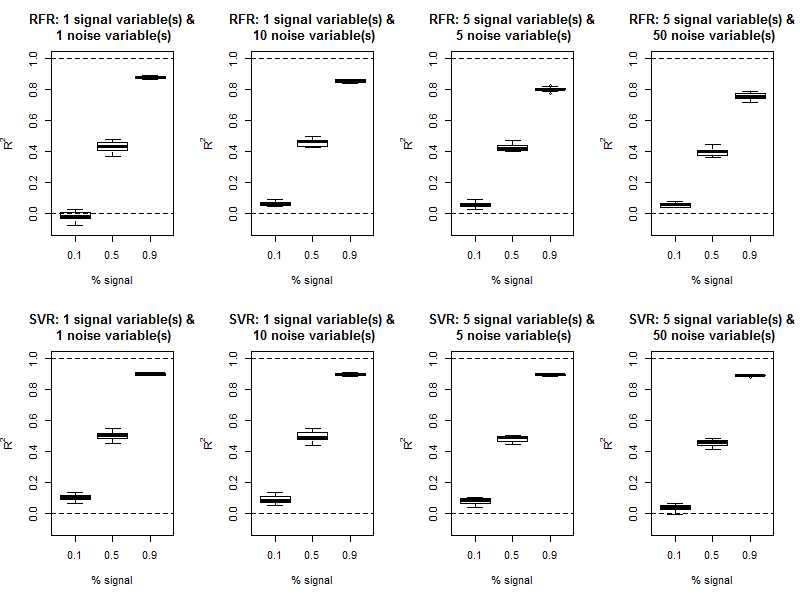


**Figure 1**: Boxplots of the explained variation (using separate train, validation and test sets) of 10 repeated simulations per combination of: algorithm (Random Forest Regression and Support Vector Regression); number of variables with a linear relationship to the response variable and number of noise variables (1/1, 1/10, 5/5, 5/50); and percentage of noise versus signal added around response variable (10%, 50%, 90%). It becomes clear that (especially SVRs) are accurately capable to predict the percentage of the response variable that contributed to the input variables until very high noise levels.
