# Supplementary material for: Inferring an animal’s environment through biologging: quantifying the environmental influence on animal movement
Source: Mov Ecol. 2020 Oct 19;8:40. doi: 10.1186/s40462-020-00228-4 (PMC7574229; doi:10.1186/s40462-020-00228-4)
Supplement: Supplementary file 2 — Additional file 2. [file 40462_2020_228_MOESM2_ESM.docx]

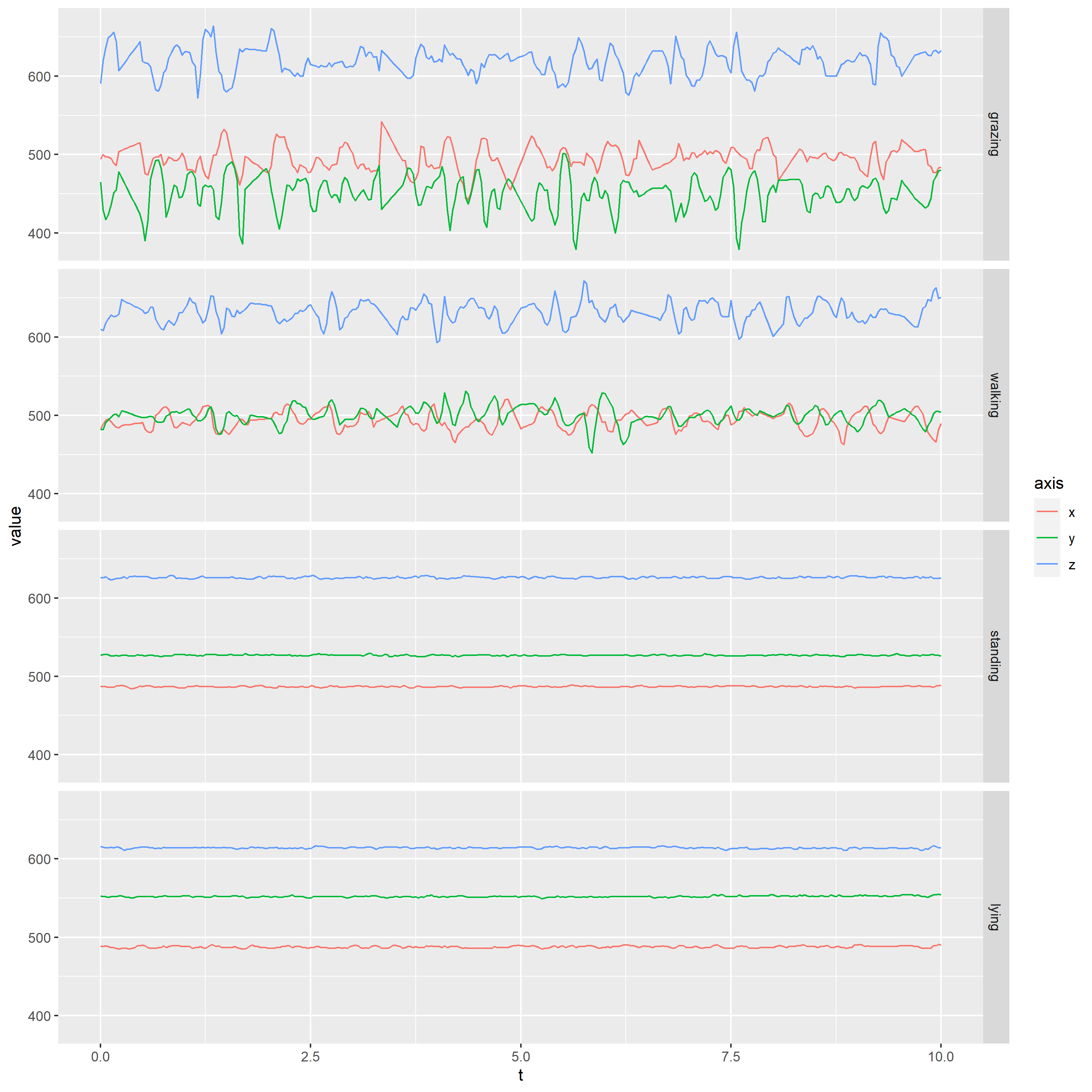


**Figure 1**: Representative acceleration plots of the three axes vs time in seconds for the different activity types.
