# Supplementary material for: Inferring an animal’s environment through biologging: quantifying the environmental influence on animal movement
Source: Mov Ecol. 2020 Oct 19;8:40. doi: 10.1186/s40462-020-00228-4 (PMC7574229; doi:10.1186/s40462-020-00228-4)
Supplement: Supplementary file 3 — Additional file 3. [file 40462_2020_228_MOESM3_ESM.docx]

We measured resource availability as dry matter grass biomass in kilograms per hectare, excluding stubble biomass. We determined time-varying biomass levels using a combination of field-measured biomass levels at specific time points, satellite-based biomass estimates derived from the Normalized Difference Vegetation Index (NDVI), and modelling of grass dynamics. The biomass field-measure was determined directly with an EC-09 electronic rising plate pasture meter (JenQuip, Feilding, New Zealand) by using the mean of 16 measurements spread equally across a pasture plot. These measurements were done roughly halfway each pasture session. For the satellite-measures, we used the Sentinel-2A (10m resolution), PlanetScope (3.125m resolution) and RapidEye (5m resolution) satellite image data to determine the NDVI inside the three pasture plots. The days for which satellite images were acquired aligned roughly with the beginning and the end of each pasture session. NDVI was calibrated against biomass using ground-truth pasture plots in and around Wageningen, Netherlands, 2016 [1].

To estimate the biomass value at any point in time during the experiment and to account for uncertainties in the measurements, we fitted an exponential decay function through the biomass measurements using numerical optimization for the decay rate coefficient (Equation 1). For this we combined the biomass measurements of the plate pasture meter and the satellite images.

$r=\frac{2000}{a_{n}}e^{-bx}+800$ (**Equation 1**),

where *r* is biomass in kilogram dry matter per hectare, excluding stubble; *a_n_* is a coefficient that determines the intercept of the function, which has a unique value for each pasture plot session; *b* is a coefficient that determines the rate of the exponential decay, which has the same value for all pasture plot sessions; and *x* is the time in days starting at zero on 12:00 PM CEST of the first day of each pasture plot session. We first determined *a_n_* for each of the three plots separately by dividing 2000 by the biomass measurement for *x* = 0. Then we multiplied all measurements from plot *n* by *a_n_* and afterwards pooled all converted measurements into one dataset. Optimizing the decay rate coefficient in the aforementioned function for this combined dataset resulted in an acceptable fit of the estimated data to the measured data (*R^2^* = 0.69; Fig. 1). These estimated data do not perfectly fit true absolute biomass levels, but nonetheless capture the trend in biomass over time.


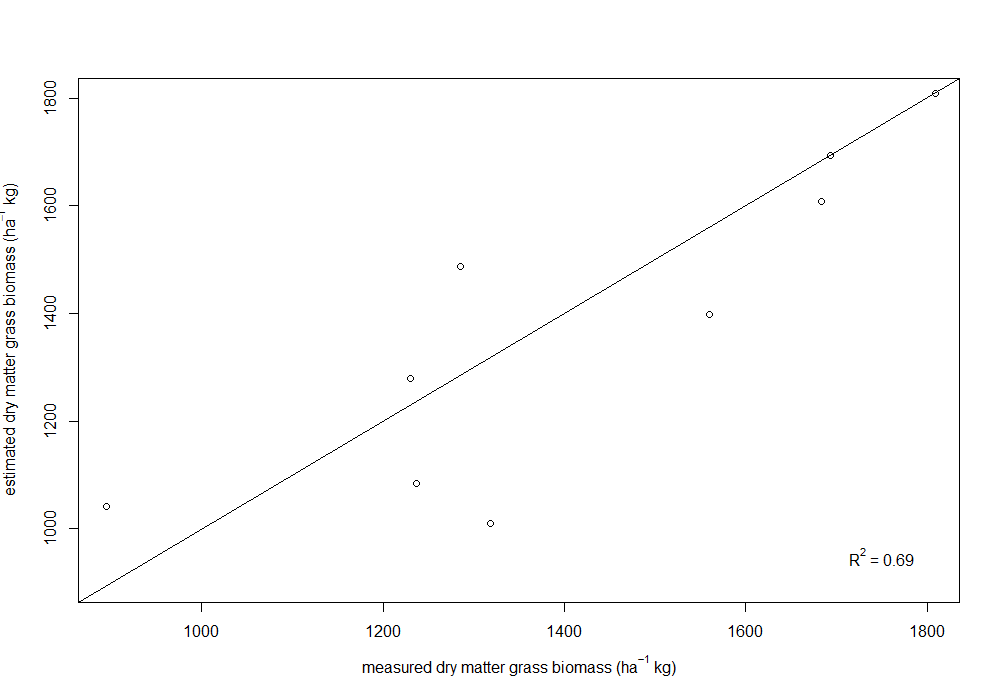


**Figure 1**: Measured versus estimated grass biomass (kilogram dry matter per hectare, excluding stubble) during the experiment.

**References**

1. Wageningen University & Research, Noldus. GrazingSpace: Final Report. Noordwijk, Netherlands; 2017.
