# Supplementary material for: Inferring an animal’s environment through biologging: quantifying the environmental influence on animal movement
Source: Mov Ecol. 2020 Oct 19;8:40. doi: 10.1186/s40462-020-00228-4 (PMC7574229; doi:10.1186/s40462-020-00228-4)
Supplement: Supplementary file 4 — Additional file 4. [file 40462_2020_228_MOESM4_ESM.docx]

**Table 1**: Hyperparameters and performance of the best performing SVR and RFR models.

| **Model** | **Response** | **Data** | **#PC ACC** | **#PC GPS** | ***cost*** | ***gamma*** | ***epsilon*** | ***R^2^*** |
| --- | --- | --- | --- | --- | --- | --- | --- | --- |
| SVR | Biomass | ACC | 29 | n.a. | 10^2.25^ | 10^-2.25^ | 0 | .29 |
| SVR | Biomass | GPS | n.a. | 6 | 10^2.25^ | 10^-1.5^ | 10^-9^ | .08 |
| SVR | Biomass | ACC+GPS | 29 | 9 | 10^2.25^ | 10^-2.25^ | 1 | .37 |
| SVR | Milk time | ACC | 21 | n.a. | 10^0.5^ | 10^-1.5^ | 1 | .21 |
| SVR | Milk time | GPS | n.a. | 7 | 10^0^ | 10^-1^ | 1 | .29 |
| SVR | Milk time | ACC+GPS | 10 | 29 | 10^0.5^ | 10^-1.75^ | 1 | .33 |
| SVR | Wind speed | ACC | 3 | n.a. | 10^1.75^ | 10^-1.25^ | 1 | -.10 |
| SVR | Wind speed | GPS | n.a. | 31 | 10^3.5^ | 10^0^ | 1 | -.12 |
| SVR | Wind speed | ACC+GPS | 3 | 0 | 10^1.75^ | 10^-1.25^ | 1 | -.10 |
| RFR | Biomass | ACC | 33 | n.a. | n.a. | n.a. | n.a. | .18 |
| RFR | Biomass | GPS | n.a. | 11 | n.a. | n.a. | n.a. | .05 |
| RFR | Biomass | ACC+GPS | 33 | 9 | n.a. | n.a. | n.a. | .19 |
| RFR | Milk time | ACC | 49 | n.a. | n.a. | n.a. | n.a. | .16 |
| RFR | Milk time | GPS | n.a. | 11 | n.a. | n.a. | n.a. | .29 |
| RFR | Milk time | ACC+GPS | 0 | 11 | n.a. | n.a. | n.a. | .29 |
| RFR | Wind speed | ACC | 122 | n.a. | n.a. | n.a. | n.a. | -.13 |
| RFR | Wind speed | GPS | n.a. | 92 | n.a. | n.a. | n.a. | -.26 |
| RFR | Wind speed | ACC+GPS | 3 | 1 | n.a. | n.a. | n.a. | -.12 |


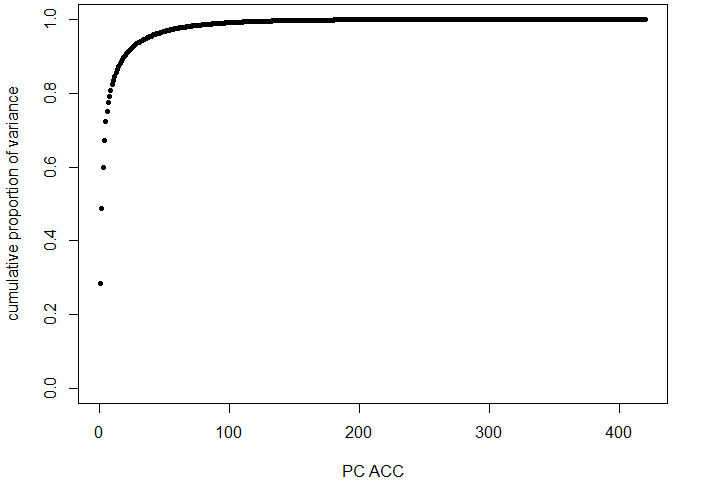


**Figure 1**: Accelerometer principal components versus cumulative proportion of variance.


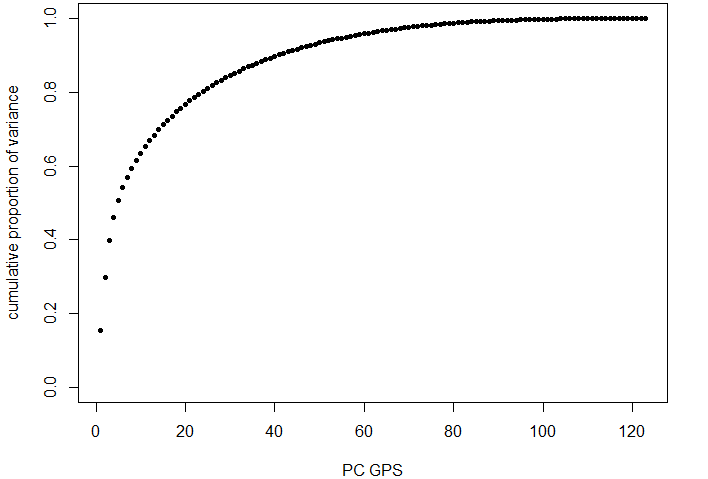


**Figure 2**: GPS principal components versus cumulative proportion of variance.
