# Supplementary material for: Inferring an animal’s environment through biologging: quantifying the environmental influence on animal movement
Source: Mov Ecol. 2020 Oct 19;8:40. doi: 10.1186/s40462-020-00228-4 (PMC7574229; doi:10.1186/s40462-020-00228-4)
Supplement: Supplementary file 5 — Additional file 5. [file 40462_2020_228_MOESM5_ESM.docx]

**Table 1**: Hyperparameters of the best performing SVM activity classification models.

|  | **Main activity types** | **Rumination** |
| --- | --- | --- |
| *window size* | 3 s | |
| *principal components* | 210 (0.999990 cum. explained variance) | 5 (0.765970 cum. explained variance) |
| *gamma* | 10^-4.7^ | 10^-2.2^ |
| *cost* | 10^3.3^ | 10^6^ |
| *class weights* | 1:1:1:1 | 1:1 |
