# Supplementary material for: Inferring an animal’s environment through biologging: quantifying the environmental influence on animal movement
Source: Mov Ecol. 2020 Oct 19;8:40. doi: 10.1186/s40462-020-00228-4 (PMC7574229; doi:10.1186/s40462-020-00228-4)
Supplement: Supplementary file 6 — Additional file 6. [file 40462_2020_228_MOESM6_ESM.docx]

**Table 1**: Confusion matrices of the best performing SVM activity classification models on the test set, for **a)** main activity types (*g = grazing; w = walking; s = standing; l = lying*); and **b)** rumination (*0 = not ruminating; 1 = ruminating*).

|  | actual | | | | | | | | | | | | |
| --- | --- | --- | --- | --- | --- | --- | --- | --- | --- | --- | --- | --- | --- |
| predicted | **a)** | *g* | *w* | *s* | *l* |  |  | |  | |  | | |
|  | *g* | **1769** | 46 | 25 | 4 |  | **b)** | *0* | | *1* | |  |  |
|  | *w* | 12 | **118** | 5 | 4 |  | *0* | **560** | | 27 | |  |  |
|  | *s* | 23 | 5 | **188** | 5 |  | *1* | 56 | | **269** | |  |  |
|  | *l* | 14 | 0 | 12 | **422** |  |  | |  | |  | | |
