# Supplementary material for: Inferring an animal’s environment through biologging: quantifying the environmental influence on animal movement
Source: Mov Ecol. 2020 Oct 19;8:40. doi: 10.1186/s40462-020-00228-4 (PMC7574229; doi:10.1186/s40462-020-00228-4)
Supplement: Supplementary file 7 — Additional file 7. [file 40462_2020_228_MOESM7_ESM.docx]

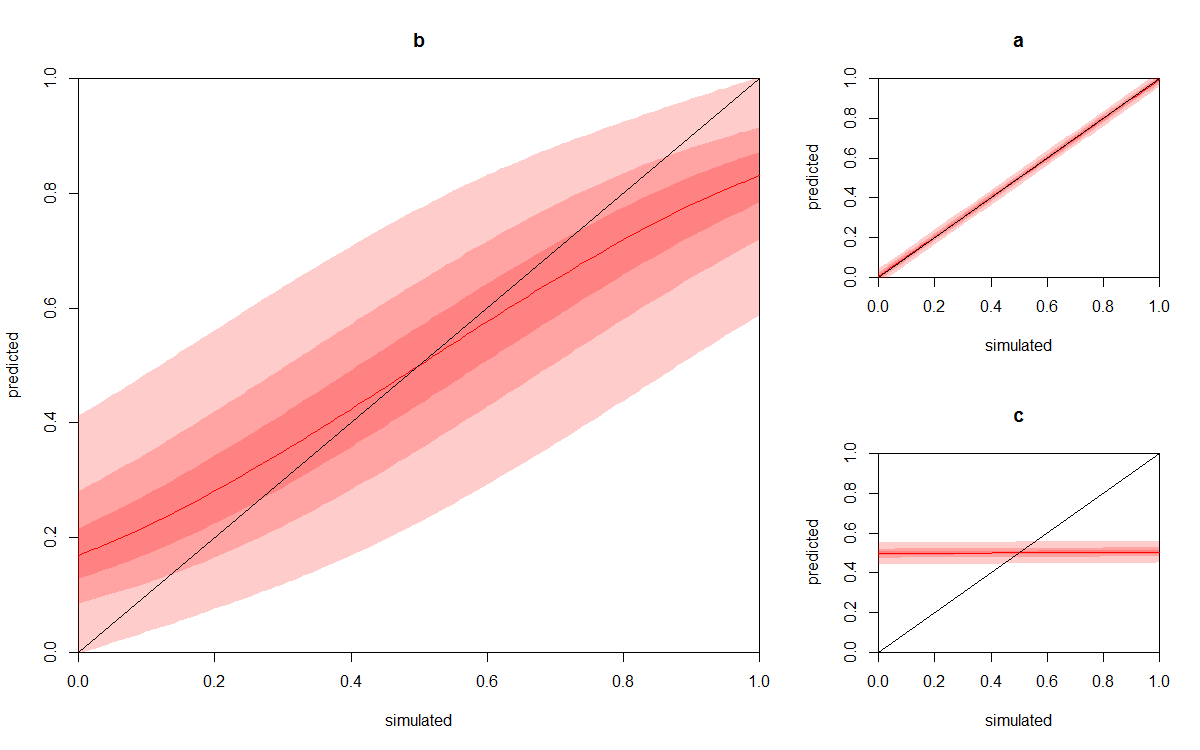


**Figure 1**: Simulated data vector ($y=\frac{\left[ 0..200 \right]}{200}$) versus the quantiles ($p=\left\{ 0.05, 0.2, 0.35, 0.5, 0.65, 0.8, 0.95 \right\}$) of 10^6^ predicted data vectors by the best performing Support Vector Regression models (Fig. 1a: *cost* = 10^4^, *gamma* = 10^-3^, *epsilon* = 10^-5^; Fig. 1b: *cost* = 10^2^, *gamma* = 10^-1^, *epsilon* = 10^-1^; Fig. 1c: *cost* = 10^1^, *gamma* = 10^-5^, *epsilon* = 10^-5^). The models were trained on the odd elements of 10^6^ different sets of two simulated input data vectors (Fig. 1a: $x_{1}\mathcal{\sim N}\left( \mu=y,\sigma=0.025 \right)$, $x_{2}\mathcal{\sim N}\left( \mu=y,\sigma=0.05 \right)$; Fig. 1b: $x_{1}\mathcal{\sim N}\left( \mu=y,\sigma=0.25 \right)$, $x_{2}\mathcal{\sim N}\left( \mu=y,\sigma=0.5 \right)$; Fig. 1c: $x_{1}\mathcal{\sim N}\left( \mu=y,\sigma=2.5 \right)$, $x_{2}\mathcal{\sim N}\left( \mu=y,\sigma=5 \right)$), and afterwards the predictions were made using the even elements of the input vectors. From these simulations it becomes clear that with a decreasing signature of the response variable in the input variables (Fig. 1a to Fig. 1c), the predictions increasingly get regressed toward the mean. In other words, the smaller the signature of the response variable in the input variables is, the more the model predictions will be located around the mean of the response variable instead of around the gradient of the response variable.
